# Research on patient-facing chatbots based on large language models in the care of older people: a living systematic review

**DOI:** 10.1007/s41999-026-01454-6

**Published:** 2026-03-13

**Authors:** Jacob T. Johnson, Jan J. Duin, Tiberon Kuiper, Yvonne M. Drewes, Jacobijn Gussekloo, Frederiek van den Bos, Armel E. J. L. Lefebvre, Marco Spruit, Bram van Dijk, Simon P. Mooijaart

**Affiliations:** 1 Department of Internal Medicine, Section of Gerontology and Geriatrics, Leiden University Medical Center, Albinusdreef 2, 2333 ZA Leiden, The Netherlands; 2 LUMC Center for Medicine for Older People, Leiden University Medical Center, Albinusdreef 2, 2333 ZA Leiden, The Netherlands; 3 Department of Public Health and Primary Care, Leiden University Medical Center, Albinusdreef 2, 2333 ZA Leiden, The Netherlands; 4 Leiden Institute of Advanced Computer Science, Leiden University, Einsteinweg 55, 2333CC Leiden, The Netherlands; 5 Department of Internal Medicine, Section of Geriatrics and Gerontology, Leiden University Medical Center, Postzone C7-Q, P.O. Box 9600 RC, Leiden, The Netherlands

**Keywords:** Older people, Chatbots, Large language models, Artificial intelligence, Natural language processing

## Abstract

**Purpose:**

Chatbots based on large language models (LLMs), like ChatGPT, have promise to augment care of older people. The evidence base, however, is small. The aim of this systematic review is to study the evidence base of patient-facing LLM chatbots in the care of older people.

**Methods:**

Following the PRISMA guidelines, a systematic search was conducted in PubMed, Embase, Web of Science, CINAHL, and Cochrane Library up to 1st May 2025 (PROSPERO—CRD42025638985). Studies involving patient-facing LLM chatbots and patients aged 60 years or older were included. Characteristics of the study and chatbots were extracted including their technology readiness levels (TRL). The mixed methods appraisal tool (MMAT) was used to assess the quality of included studies.

**Results:**

Out of 1228 records, 9 studies were included, with a median sample size of 12 older participants. Of these, 5 were mixed methods, 3 were qualitative, and 1 was a pilot/feasibility study. Seven studies evaluated chatbots providing supportive conversations or social/emotional support. GPT-3.5 (OpenAI) was the most frequently employed LLM. Median TRL was 7; no LLM chatbot was assessed at a TRL of 8 or higher (tested for effectiveness).

**Conclusions:**

Few studies report on patient-facing LLM chatbots in the care of older people. Most studies utilized qualitative methods. No study evaluated the clinical or cost-effectiveness of chatbots, and there were no studies conducted while the chatbot was fully implemented within clinical workflows. This indicates the research is still in its early exploratory phases. This living review will be updated periodically to follow developments in the field.

## Key Summary Points

**Aim:**

The aim of this systematic review is to study the emerging evidence base on patient-facing LLM chatbots in the care of older people.

**Findings:**

This systematic review identified nine studies on patient-facing LLM chatbots for the care of older people, finding that most used qualitative research and no study reported on their effectiveness while fully operational.

**Message:**

Research in the field of LLM chatbots used in the care of older people is still in its early phases.

## Introduction

Chatbots powered by large language models (LLMs) like ChatGPT are increasingly being used in everyday life and are entering the healthcare domain, but research on their use is still scarce. The rising number of older people combined with a shortage of healthcare staff creates increasing pressure on care access and delivery. Patient-facing LLM chatbots are being explored and developed to support older adults across practical, clinical, and well-being needs (medication reminders, disease management, companionship, geriatric assessment and goals-of-care discussions, and mental health/fitness coaching). These types of chatbots may ease clinician workload and broaden access where professionals are scarce or distant [1–4]. Any form of a LLM chatbot that directly interacts with a non-healthcare worker in a care setting we term patient-facing chatbot. This term denotes a contrast to provider-facing, healthcare personnel-facing, or caregiver-facing chatbots in which the chatbot is not meant or designed to interact with patients.

An LLM is a type of artificial intelligence that in its so-called pre-training phase learns sophisticated linguistic patterns by predicting words from context using large amounts of text, typically from the web. LLMs used to power chatbots can respond to open-ended questions and have natural, human-like conversations based on current and earlier context (such as conversation history). Studies show that they can deliver high-quality, versatile, and empathetic responses in conversations [5]. Since the introduction of ChatGPT in 2022, more and more studies report on their use in healthcare contexts [6]. Yet, translating the potential of LLM chatbots into geriatric clinical practice requires systematic evaluation in studies involving older adults, for instance to study specific requirements for older people to interact with the chatbot or the medium it is on (tablet or phone) or because the language of older people may be different in terms of tone, speed, or vocabulary. These areas are not often explored [7–9]. A wider range of studies on the development of this technology is needed before successful implementation, such as on their feasibility, usability, and effectiveness in older people [10]. While these tools are promising, like assisting in geriatric assessment, an overview of their evidence base in the care of older people is missing.

The aim of this systematic review is to study the evidence base of patient-facing LLM chatbots in the care of older people.

## Methods

### Baseline living review

This study will follow a living systematic review approach. A living review approach is recommended when evidence is rapidly expanding, when new findings may alter conclusions, and when decision makers require reliable summaries to guide practice and policy. Living reviews also ensure timeliness by updating findings in smaller, ongoing steps rather than through infrequent large updates, which is particularly important as clinical evaluations of LLM chatbots increase. Furthermore, performing pre-planned updates allows for robust study of developments in the field. A baseline systematic review is needed for studying LLM chatbots because the field is developing quickly and the current evidence is likely limited and varied for this field. This review follows the guidelines of the International Living Systematic Review Network [11] and establishes the foundation for examining LLM chatbots in the care of older people. It defines inclusion criteria, outcomes, and analytic methods, providing a reference point for a living review that can be updated as new studies emerge. As new studies are published, they will be identified through database searches and added to the evidence base following the same inclusion and analysis procedures. We plan to do this two years from now. This update schedule and decision framework are based on published methodological guidance for optimizing and updating living systematic reviews (Journal of Clinical Epidemiology, [12]). We selected a 2-year update interval so that new evidence is more likely to be relevant beyond pilot studies and worth reanalyzing rather than just appended. This cadence is also aligned with real-world product development timelines for this field. At each 2-year search update, to determine whether the new evidence warrants a major revision we will use these triggers (i), if the new evidence fundamentally alters previously stated conclusions, (ii), the volume of new evidence sufficiently outnumbers previous versions, (iii), new methodological approaches are defined such as quality appraisal tools specific to this field of research (iv), stakeholder input indicates that the current evidence base is no longer sufficient for decision making. For example, if there are sufficient studies conducted under real-world clinical conditions for clinical and cost-effectiveness or if the chatbots are being studied while fully implemented in the care pathway. If these signals are not met, the update will be limited to an evidence refresh (study inventory and descriptive mapping) without revising the main conclusions. If changes are warranted, these will be documented through a protocol amendment with rationale and date. A transparent section on what is new will be added.

### Search strategy

This systematic review was conducted following the PRISMA guidelines (http://www.prisma-statement.org/) for reporting and design of systematic reviews (Supplementary Sect. 1). Registration was performed on PROSPERO registration number CRD42025638985. A systematic query was conducted in the databases of PubMed, Embase, Web of Science, CINAHL, and Cochrane Library with the assistance of a trained librarian (T.K.P.). Databases were searched from 2018 (OpenAI released the first iteration of GPT, known as GPT-1 in this year, although this early model was mainly a technical tool for tasks like sentence similarity and not a general conversational system) up to 1st May 2025 [13]. Date limits were not embedded in the search strings. Instead, we applied a publication date filter (January 1, 2018 onward) in each database interface after running the search. Searches were conducted using a combination of keywords related to chatbots, LLMs, geriatrics, and terms associated with care of older adults. The complete search strategy can be found in Supplementary Sect. 2. We utilized ASReview for the title and abstract screening process. ASReview is an open-source software that applies machine learning to support systematic screening of large text datasets. It is commonly used for title and abstract screening in systematic reviews and meta-analyses, but can be applied to other structured text screening tasks as well [14]. For the full-text screening, Rayyan, an online literature screening tool was used [15].

### Eligibility criteria

The inclusion criteria for the studies included in the review were as follows: (1) All patients in the study population or specific subgroup of patients must be 60 years or older. (2) The study must have included a chatbot that employed an LLM. (3) Studies had to provide sufficient data to answer our research question, meaning they reported the chatbot architecture, the role of the LLM within it, the study population, and the research question. (4) Studies must have focused on the health, well-being, or lifestyle of older people, where the participant directly interacted with the chatbot, hence patient-facing. (5) The article must have been published in English. The exclusion criteria were (1) studies registered as editorials, letters, reviews, or case reports. (2) Studies were excluded if the chatbot type was unclear or not specified as using an LLM. (3) Studies were excluded if the study used the chatbot for purposes unrelated to the medical field, such as a chatbot assisting participants in following the news. The selected search strategy yielded studies, which were screened by one author (J.T.J.) on title and abstract. ASReview was used, but the author ended up screening all articles before reaching the point of automatic sorting by the software. Consequently, a full-text assessment of the screened articles was independently performed by 3 authors (J.T.J., J.J.D., and S.P.M.). Each article was assessed by 2 of the 3 authors. In case of disagreement about inclusion, the third author was involved to make the final decision.

### Data extraction

Three authors (J.T.J., J.J.D., and S.P.M.) independently extracted data from the selected records into a Microsoft Excel spreadsheet. For each included study, we recorded the following information: first author, date of publication, journal name, country where the study was conducted, study type, age criteria, reported age of participants (specifying mean, median, or range), number of participants or sample size, target population (e.g., patients, caregivers), study setting (e.g. at home, nursing home or multi-setting), and type of intervention (e.g., supportive conversation, educational, diagnostic). Additionally, we documented the outcomes measured (e.g., acceptability, usability, cultural relevance), the name of the LLM used (if specified), whether the design of the user interaction was tailored for older adults (e.g., adjustment to audio speed, larger text display, researcher present in the interaction with the older adult), information on the platform used (e.g., web-based application, smartphone-based app), input modality (e.g., text, voice), output modality (e.g., text, voice, video); these categories were chosen based on prior work by Zhang et al. [8]. We classified the studies according to the Technology Readiness Level (TRL), which are used to indicate phases of innovation and product development [16]. TRLs offer a standardized way to measure how developed a technology is, guiding its path from early concept to practical application. The TRL is interpreted by a score from 1 to 9, where a lower number is less developed and a higher number is more developed. First introduced by NASA, the TRL scale is now used across sectors, though its meaning and application can differ by field. In our review, we adapted the’General Description’ of TRLs to our ‘Specific Description’ (see Table 1). In our adaptation of the development phases for LLM chatbots in the care of older people, TRL 1 represents the beginning research phase, while TRL 9 represents a chatbot that is fully implemented into clinical care. As TRL was not reported in any of the studies, each author (J.T.J., J.J.D., S.P.M.) independently assessed the TRL level of the studies’ use of LLM chatbots, after which differences were discussed until all three authors (J.T.J., J.J.D., and S.P.M.) reached consensus on the final categorization.

**Table 1:** Technology readiness levels (TRL)

TRL (#)	General description	Specific description
1	Scientific research begins; basic concepts and principles are being explored	Scientific research begins; basic concepts and principles are being explored
2	Invention and application are formulated; potential practical applications are identified	Description of clinical target group, context and intended purpose
3	Laboratory-based testing to validate the feasibility of the concept	Testing of the chatbot elements by study personnel
4	Components and systems are integrated and tested in a controlled laboratory setting	Prototype chatbot tested by study personnel or other volunteers for usability and feasibility
5	Testing moves beyond the lab into a simulated or relevant environment	Prototype chatbot tested by older people for usability and feasibility
6	Prototype or system is demonstrated in an operational or relevant environment	Prototype chatbot tested by older people for usability and feasibility in the relevant context
7	Demonstration of the system in the intended operational environment, showing functionality	Chatbot tested by older people in the relevant context for its intended purpose
8	The system is proven to work and is qualified through rigorous testing and evaluation	Effectiveness and cost-effectiveness tested in clinical trial (such as RCT)
9	The technology is fully deployed, operational, and used successfully in its intended environment	Chatbot implemented in clinical care

Table 1 shows the 'General Description' and 'Specific Description' columns where we describe our adaptation of the phases of development for LLM chatbots used in the care of older people. TRLs are used to indicate phases of innovation and product development

### Quality assessment

Each study was appraised for study quality independently by the authors (J.T.J., J.J.D., and S.P.M.) using the Mixed Method Assessment Tool [17]. This tool first uses two brief general screening questions to assess if the article is suitable to be assessed using this tool; the tool then applies specific criteria depending on the category of study (qualitative, quantitative randomized controlled trials, quantitative non-randomized, quantitative descriptive, and mixed methods). This tool was chosen since it is suitable to assess the diverse study types included in this review. To our knowledge, there are no quality assessment criteria made to evaluate LLM chatbot studies; therefore, this tool was chosen in proxy. The quality of each study was assessed by 2 of the 3 authors, and in case of disagreement between authors, a third author was involved to make the final decision. Quality assessments are presented per study since reporting summary statistics based on this tool is discouraged (Supplementary Sect. 3).

## Results

**Fig. 1:**
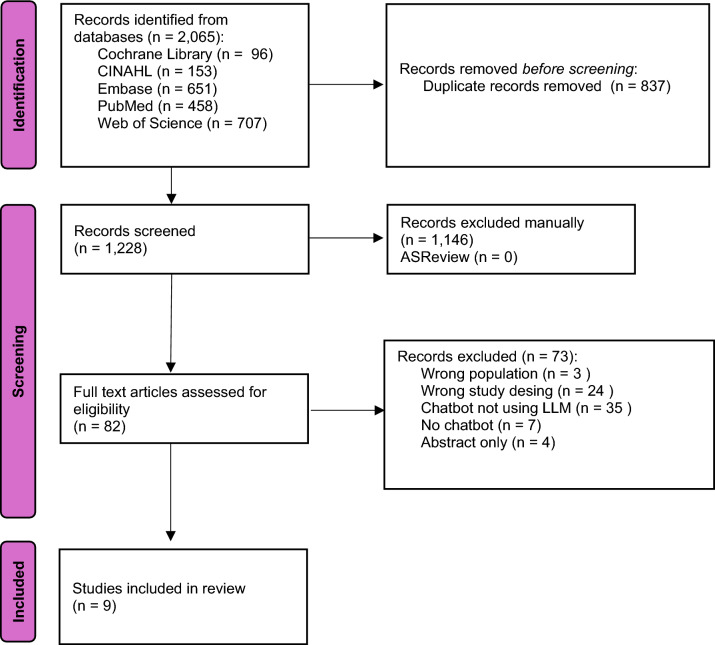
PRISMA flow diagram

The flowchart of the study selection process is presented in Fig. 1. Initially, our search yielded 2,065 records, which were subsequently reduced to 1,228 after the removal of duplicates. These records underwent screening with ASReview based on title and abstract, leading to the assessment of 82 articles for eligibility through full-text review. Following the comprehensive full screening evaluation, 73 articles were excluded, resulting in a final inclusion of 9 articles in our review. Reasons for exclusion are mentioned in Fig. 1.

Table 2 summarizes the characteristics of the nine included studies. Sample sizes ranged from 8 to 28 participants, with a median of 12. Mean of the mean participant age was 74.6 years, with a mean standard deviation of 5.9 years. Geographically, studies were conducted in the United States (4 studies) [3, 18–20], followed by Japan (2 studies) [21, 22]. The remaining studies were conducted in Sweden [23], Canada [24], and China [25]. Mixed methods design was used in five studies, qualitative research in three studies, and one study was a pilot/feasibility study. The most frequent study setting was home environments (5 studies), followed by nursing homes and studies using a variety of settings (2 studies each). Interventions primarily involved supportive conversations and social/emotional support (7 studies) of older people, followed by patient education (3 studies). Several studies addressed multiple intervention types (e.g., social/emotional support and patient education). Other interventions focused on diagnostic support (e.g., collecting data for a geriatric assessment), caregiver education (e.g., information resource on caring for persons living with dementia), behavior change (e.g., motivational coach for exercise), and patient-provider communication (e.g., chatbot was used to assist people on the phone). Seven studies reported more than one outcome. The reported outcomes were related to early-stage feasibility, usability, acceptability, and patient-reported measures (loneliness, cultural relevance, depression, life satisfaction, communication quality, and user expectations/preferences). No study reported objective clinical outcomes related to geriatric assessment, safety, healthcare utilization, or cost-effectiveness in a real-world setting.

**Table 2:** Study characteristics

	First author	Year	Study design	Type of intervention	Sample size	Reported age of participants (mean[SD])	Target population	Outcomes measured	Country of study	Setting
1	Al Mazroui, K	2024	Mixed Methods	Supportive conversations, Patient education, Social/emotional support	20	65.3 (3.4)	Older adults	Loneliness	US	Multi setting
2	Bosco, C	2025	Qualitative	Caregiver education	15	68.1 (5.28)	Caregivers of people living with dementia	Acceptability, Usability, Cultural relevance, Adoption	US	Home
3	Browne, R	2024	Mixed Methods	Behavior change, Supportive conversations, Patient education	8	70.85 (6.0)	Older people in general	Acceptability, Usability	Japan	Home
4	Cuadra, A	2024	Mixed Methods	Diagnostic support	10	80 (13.44)	Patients	Acceptability, Usability	US	Multi setting
5	Irfan, B	2024	Qualitative	Supportive conversations, Social/emotional support	28	74.5 (5.6)	Older adults	Acceptability, Usability, Expectations and preferences	Sweden	Home
6	Sheehy, L	2024	Qualitative	Supportive conversations, Social/emotional support	20	81 (6.9)	People living with dementia	Feasibility, Acceptability	Canada	Nursing Home
7	Shimizu, K	2024	Pilot/feasibility study	Supportive conversations, Social/emotional support	12	Not available	Healthy older adults	Effectiveness	Japan	Community dwelling (neighborhood resource center)
8	Wang, Y	2024	Mixed Methods	Social/emotional support	12	79,5 (1.3)	Older adults in nursing homes	Usability, Loneliness, Depression, Life satisfaction	China	Nursing Home
9	Yang, Z	2024	Mixed Methods	Patient-provider communication	10	77.5 (6)	Older adults (patients) and healthcare providers	Usability, Communication quality	US	At home (older adults) and clinical settings (providers)

Table 2 includes details regarding study design, intervention types, sample sizes, reported ages, target populations, measured outcomes, country of study, and study settings

**Fig. 2:**
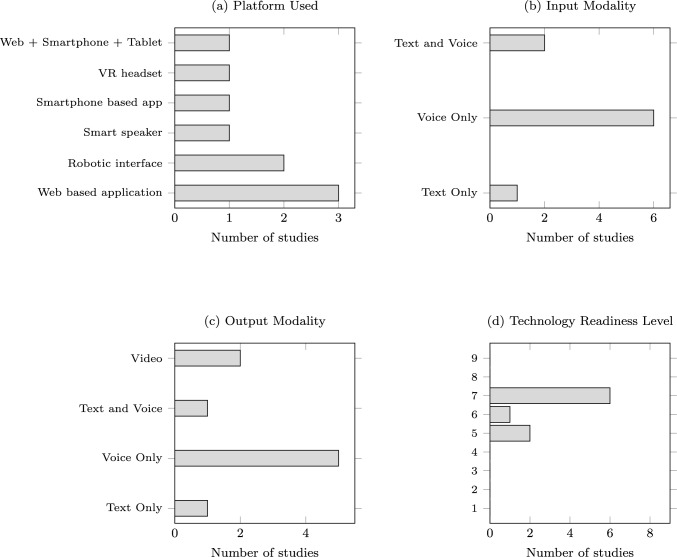
Chatbot characteristics in the included studies: **a** Platform used, **b** Input modality, **c** Output modality, **d** TRL

Table 3 describes the LLM chatbot characteristics used in the included studies. Various platforms were utilized to deploy chatbots: web-based applications (3 studies), smartphone-based apps (2 studies), a virtual reality headset (1 study), LLM adapted smart speaker (1 study), and a computer-based interface (1 study), robotic interfaces (1 study). Some studies employed multiple platforms. Regarding input modalities, voice was the most frequently utilized (8 studies). Text was employed in three studies. Two studies supported touch-based interaction: one provided a standard touchscreen app mode alongside text chat and voice, letting users tap to navigate and select content. The other used on-screen images and buttons that participants could tap, including options to play an audio rendering of the chatbot’s reply. Voice was the universal output modality across all studies (100%), often used in combination with other modalities such as text (3 studies), video (2 studies), and images (1 study). Seven studies explicitly indicated that the user interface was specifically built or adapted for older adults, for example, adjustment to speech speed, while three studies did not report whether specific adaptations were made. The studies identified the LLM used, with ChatGPT 3.5 Turbo being the most frequent (4 studies), followed by GPT-3.5, GPT-4, and text-davinci-003 (1 study each). GPT 3.5 and its variants are proprietary and cannot be hosted locally; it is also not known when updates occurred [26]. The median TRL score of the LLM chatbots used in the studies in this review was TRL 7, meaning the chatbot was tested in a study environment by older people in its relevant context for its intended purpose. No study reported on the LLM chatbots’ effectiveness while being fully operational in their intended environment (TRL 8–9). The median TRL score of the included studies was TRL 7, indicating that while these chatbots were functional and tested by older adults within their intended environments—for example, at home or in nursing homes—they have not yet reached the stage of rigorous effectiveness testing. Notably, no studies met the criteria for TRL 8 or 9, which would require a transition from feasibility testing to formal clinical trials or full implementation into standard clinical care (Fig. 2).

**Table 3:** Chatbot characteristics

	First author	Type of intervention	Technology Readiness Level (TRL)	Outcomes measured	Platform used	Input Modality	Output Modality	Name of large language model used (if there is a name)	Was modality built or adapted for older people?
1	Al Mazroui, K	Supportive conversations, Patient education, Social/emotional support	TRL 7	Loneliness	Web based application	Text	Text	ChatGPT (version not specified)	No
2	Bosco, C	Caregiver education	TRL 7	Acceptability, Usability, Cultural relevance, Adoption	Smartphone based app	Text, Voice, Touch	Text, Voice	ChatGPT 3.5 Turbo	Yes
3	Browne, R	Behavior change, Supportive conversations, Patient education	TRL 7	Acceptability, Usability	Robotic interface	Voice	Voice	GPT-3.5-Turbo	No
4	Cuadra, A	Diagnostic support	TRL 7	Acceptability, Usability	Web based application, Smartphone based app, Tablet based app	Text, Voice, Touch	Text, Voice, Video	GPT-4	Yes
5	Irfan, B	Supportive conversations, Social/emotional support	TRL 5	Acceptability, Usability, Expectations and preferences	Robotic interface	Voice	Voice	GPT-3.5 text- davinci-003	Yes
6	Sheehy, L	Supportive conversations, Social/emotional support	TRL 6	Feasibility, Acceptability	VR headset	Voice	Voice, Video	GPT-3.5	Yes
7	Shimizu, K	Supportive conversations, Social/emotional support	TRL 5	Effectiveness	Web based application	Voice	Voice	GPT-3.5-turbo	Yes
8	Wang, Y	Social/emotional support	TRL 7	Usability, Loneliness, Depression, Life satisfaction	Web based application	Voice	Voice	ChatGPT 3.0	Yes
9	Yang, Z	Patient-provider communication	TRL 7	Usability, Communication quality	Smart speaker	Voice	Voice	GPT-3.5-Turbo	No

Table 3 provides an overview of the chatbot characteristics employed across the studies. This includes the type of intervention, technology readiness level (TRL), platform, input and output modalities, specific large language models (LLMs) utilized, and whether these chatbot modalities were specifically built or adapted for older adults

The quality of the studies, assessed using the Mixed Methods Appraisal Tool (MMAT), is detailed in Supplementary Sect. 3. Of the nine included studies, two had a qualitative design, both of which met all five MMAT quality criteria, which according to this tool, meant their quality was high. Among the seven mixed-methods studies, available reporting varied. Four of the seven mixed-method studies provided sufficient information to assess both qualitative and quantitative components, while others lacked detail to confidently evaluate several criteria. Overall, the included studies demonstrated adequate alignment between methods and research questions; issues with reporting and limited detail were common, particularly in the quantitative portions of mixed-method designs.

## Discussion

This systematic review identified nine peer-reviewed international scientific studies on patient-facing LLM chatbots in the care of older people. Most of these studies utilized qualitative research. No study reported studying the LLM chatbot for its clinical or cost-effectiveness or while being fully operational in its intended environment (TRL 8–9).

As far as we know, this study is the first systematic review that has specifically focused on patient-facing LLM chatbots in the care of older people. A scoping review by Zhang et al. studied chatbots in the care of older people with searches up to May 2023, but it did not focus specifically on studies using LLM chatbots [8]. Zhang et al. included 29 articles, five of which used Natural Language Processing (NLP) chatbots. NLP however subsumes work on LLMs, but is a broader research field, and the chatbots in their review did not incorporate LLMs. In contrast, our review identified nine studies explicitly using LLMs, none of which were included in theirs. Zhang et al. found most chatbots were used for well-being, cognitive training, and health information which aligns with An et al.’s preprint review though they did not specify LLM use (available at arXiv:2503.23153).

While there are already LLM chatbots being used in other hospital settings such as scribes to record outpatient clinic visits and in patient-provider communication portals [6, 27, 28], the quantities of studies on their use in the care of older people remain limited [7]. Most existing studies utilize qualitative methods, are conducted in non-clinical settings, and have not tested for clinical effectiveness or while fully operational (TRL 8–9) [29, 30]. Another reason might be that clinical studies on emerging technologies typically lag behind commercial development due to the time required for study design, ethics approval, and the publication process [31]. From a geriatric perspective, using patient-facing LLM chatbots within care pathways raises specific concerns about ethics and safety [32]. Sensory impairments change older patients’ ability to engage with chatbots which limit the accessibility of them [33]. Older patients with cognitive decline are more vulnerable to misinformation and may have reduced awareness of data sharing practices or limited capacity to manage consent [34]. Dependence on conversational agents for health information or emotional support may further exacerbate risks related to autonomy, safety, and delayed professional care [35]. Further, older people are often underrepresented in testing healthcare innovations, even though they are frequent users of healthcare [36]. There are also inherent risks with this technology which could lead to unintended negative consequences in vulnerable populations, including data privacy concerns, potential misinformation, and biases (such as ageism) embedded in training data of LLMs [37].

The results of this study show that research on patient-facing LLM chatbots is a developing field. The focus of current studies is on prototype development and early-stage feasibility, usability, acceptability, and patient-reported outcomes. This is in contrast to studies that would fit into TRL level classification which would report outcomes related to geriatric assessment, safety, healthcare utilization, or cost-effectiveness. Our findings highlight that the current research field is focused on the demonstration phase (TRL 7) rather than the validation phase (TRL 8–9). The distinction between studies at TRL 7 shows that a chatbot can be used in a relevant context, whereas TRL 8 and 9 are reserved for technologies proven to be clinically and cost-effectively superior to current standards of care. Without this transition to higher readiness levels, the evidence remains insufficient to support the widespread adoption of patient-facing LLMs in geriatric practice. To move beyond this demonstration phase, future research should focus on formal implementation studies and randomized controlled trials. These studies should specifically assess implementation effects on both patient and professional satisfaction, alongside objective measures of clinical time utilization and workflow integration. Clinician and caregiver involvement throughout all stages of development, from early research (TRL 1) to advanced testing (TRL 8) helps with usability and acceptance, which may be the way to establish the missing evidence [38]. With the rapid development of LLM chatbots in society and their easy integration in commercial products such as smart speakers (Google Home, Alexa) or in devices such as robotics, we expect a surge of interest in their use in the coming years [39].

This systematic review has several strengths and limitations. One limitation is that there are no standardized quality criteria used in research to assess studies of LLM chatbots. To surpass this limitation, we used a valid mixed-methods tool to assess each study’s quality. Additionally, the categorization of study types, outcome measures, and Technology Readiness Levels (TRLs) is subjective, potentially influencing result interpretation. In healthcare research, TRL classification lacks a universal standard, requiring subjective judgment to align clinical study designs with engineering phases. To mitigate this, TRL levels in this review were independently assessed by three authors followed by a formal consensus procedure to reconcile differences. This triangulated approach was used to ensure the reliability of the innovative application of the TRL classification to the heterogeneous LLM chatbot evidence base. Finally, the review included only studies published in English, potentially missing relevant literature published in other languages. A primary strength of this review is that it is the first systematic review explicitly focused on LLM chatbots used specifically in the care of older adults. A second strength is that this review has been done in accordance with PRISMA guidelines. A third strength is that this review has systematically qualified TRL levels by two independent reviewers. Finally, we have designed this review as a baseline living systematic review so that we can do it again in two years to assess the development of this field.

To conclude, only a few studies report on patient-facing LLM chatbots in the care of older people. Most studies utilized qualitative methods. No study evaluated the clinical or cost-effectiveness of the chatbot, and there were no studies conducted while the chatbot was fully implemented within clinical workflows. This indicates the research is still in its early exploratory phases. This living review will be updated to follow developments in the field.

## Data Availability

The search strategy is available in the appendix, and any additional data are available on reasonable request to the corresponding author.
